# The UbL-UBA Ubiquilin4 protein functions as a tumor suppressor in gastric cancer by p53-dependent and p53-independent regulation of p21

**DOI:** 10.1038/s41418-018-0141-4

**Published:** 2018-06-13

**Authors:** Shengkai Huang, Yan Li, Xinghua Yuan, Mei Zhao, Jia Wang, You Li, Yuan Li, Hong Lin, Qiao Zhang, Wenjie Wang, Dongdong Li, Xin Dong, Lanfen Li, Min Liu, Weiyan Huang, Changzhi Huang

**Affiliations:** 1 State Key Laboratory of Molecular Oncology, National Cancer Center/Cancer Hospital, Chinese Academy of Medical Sciences and Peking Union Medical College, 100021 Beijing, China; 2 Department of Etiology and Carcinogenesis, National Cancer Center/Cancer Hospital, Chinese Academy of Medical Sciences and Peking Union Medical College, 100021 Beijing, China; 3 Department of Clinical Laboratory, National Cancer Center/Cancer Hospital, Chinese Academy of Medical Sciences and Peking Union Medical College, 100021 Beijing, China; 4 Beijing Key Laboratory for Carcinogenesis and Cancer Prevention, 100021 Beijing, China; 5 Department of Abdomen Surgery, National Cancer Center/Cancer Hospital, Chinese Academy of Medical Sciences and Peking Union Medical College, 100021 Beijing, China; 6 Department of Clinical Laboratory, Meitan General Hospital, 100021 Beijing, China; 7 State Key Laboratory of Cellular Stress Biology, School of Life Sciences, Xiamen University, 361005 Fujian, China; 8 Biomolecular Interaction Centre, University of Canterbury, Christchurch, 8140 New Zealand; 9 Department of Chemistry, University of Canterbury, Christchurch, 8140 New Zealand; 10 Department of Developmental Biology, China Medical University, 110122 Shenyang, China; 11 State Key Laboratory of Protein and Plant Gene Research, School of Life Sciences, Peking University, 100871 Beijing, China; 12 Department of Veterinary Biosciences, Ohio State University, Columbus, OH 43210 USA

**Keywords:** Tumour-suppressor proteins, Ubiquitin ligases

## Abstract

Ubiquilin4 (Ubqln4), a member of the UbL-UBA protein family, serves as an adaptor in the degradation of specific substrates via the proteasomal pathway. However, the biological function of Ubqln4 remains largely unknown, especially in cancer. Here, we reported that Ubqln4 was downregulated in gastric cancer tissues and functioned as a tumor suppressor by inhibiting gastric cancer cell proliferation in vivo and in vitro. Overexpression of Ubqln4-induced cellular senescence and G1-S cell cycle arrest in gastric cancer cells and activated the p53/p21 axis. Moreover, Ubqln4 regulated p21 through both p53-dependent and p53-independent manners. Ubqln4 interacted with RNF114, an E3 ubiquitin ligase of p21, and negatively regulated its expression level, which in turn stabilized p21 by attenuating proteasomal degradation of p21. These effects of Ubqln4 were partly abrogated in gastric cancer cells upon silencing of p21. Our findings not only establish the anti-tumor potential of Ubqln4 in gastric cancer but also reveal a role for Ubqln4 in regulation of the cell cycle and cellular senescence via stabilizing p21.

## Introduction

Gastric cancer (GC) is the fifth most common cancer and the third main cause of cancer mortality [1]. Systemic chemotherapy is the standard treatment for patients with advanced GC; however, the effectiveness is low, with a median survival of 6–11 months [2]. Alterations in several genes, including *ERBB2*, *PTEN*, *FGF*, *TP53*, *CDH1* and *cMET* genes, have been reported in GC, and a few are being pursued in the clinic [1]. However, better clarification of the underlying molecular mechanisms of GC can help guide new drug discovery.

Ubqlns (Ubqln1–5 and UbqlnL) belong to the UbL-UBA protein family, which show diverse biological functions in protein degradation [3–5] and nucleotide excision repair (NER) [6]. Ubqlns contain an N-terminal ubiquitin-like (UbL) domain and a C-terminal ubiquitin-associated (UBA) domain. Some studies proposed a shuttle-factor method of function for UbL-UBAs, in which UbL-UBA proteins bind ubiquitinated proteins and the proteasome via the UBA and UbL domains, respectively [7]. These proteins can thus facilitate or reduce protein degradation depending on interactions with different substrates [7] and also participate in proteasomal degradation [4, 8–10]. The Ubqln substrates show great diversity and impact a wide range of cellular functions. Ubqln1 displays anti-apoptotic potential in lung cancer cells by stabilizing Bcl-B, a Bcl-2 family protein [11]; Ubqln2 increases p53 levels by interfering with ubiquitin-mediated degradation of p53 in a UBA domain-dependent manner [4, 9]. Increasing research has uncovered roles for Ubqlns in human cancer. The Ubqln1 gene is lost or under-expressed in many human cancer cell lines [12], and Ubqln1 was reported to be involved in many types of cancers, including breast cancer [13] and lung cancer [12]. In addition, loss of Ubqln1 or Ubqln2 can induce migration, invasion, and epithelial–mesenchymal transition in non-small lung cancer cells [12].

Ubqln4 exhibits common properties of Ubqlns [14] and acts as an adapter that recruits Ubqln1 to the autophagy machinery. The direct association between Ubqln4 and protein light chain 3, an autophagosomal marker, is essential for the maturation of autophagosomes to autolysosomes by mediating autophagosome–lysosome fusion [15]. Ubqln4 is also indispensable for the interaction between the proteasome and connexin43 (Cx43), which is critical for gap junction intercellular communication. Dysregulation of Cx43 and gap junction intercellular communication is involved in several human diseases, such as cancer [16] and heart disease [17]. Ubqln4 also links ataxin-1 to the ubiquitin-proteasome pathway in spinocerebellar ataxia type 1 [18]. Together this shows that Ubqlns may have critical roles in human disease.

p21, a member of the CIP/Kip family of cyclin-dependent kinases, is a well-known cell cycle inhibitor that induces cell cycle arrest at the G1/S transition by inhibition of CDK4, 6/cyclin D [19, 20]. The level of p21 is determined by multiple mechanisms at the transcriptional, translational and posttranslational levels [21, 22]. p21 is transcriptionally regulated by p53 [23] and can also be regulated in p53-independent way [24]. Several E3 ubiquitin ligase complexes, such as RNF114 [25], SCFSkp2 [26], and MKRN1 [27] negatively regulate p21 stability by triggering p21 ubiquitination and subsequent degradation. p21 can also be stabilized by interactions with proteins that prevent its ubiquitin-independent degradation [28, 29].

Here we examined the biological function and mechanisms of Ubqln4 in GC. We demonstrated that Ubqln4 was downregulated in human GC tissues and inhibited tumorigenesis in vitro and in vivo. Ubqln4 regulated p21 levels not only through p53-dependent mechanisms, but also through a p53-independent pathway by binding to RNF114, an E3 ubiquitin ligase of p21. Our study establishes a novel tumor-suppressive function for Ubqln4 in GC that involves regulation of p21 through multiple pathways.

## Results

### Ubqln4 expression is significantly decreased in GC tissues

**Fig. 1:**
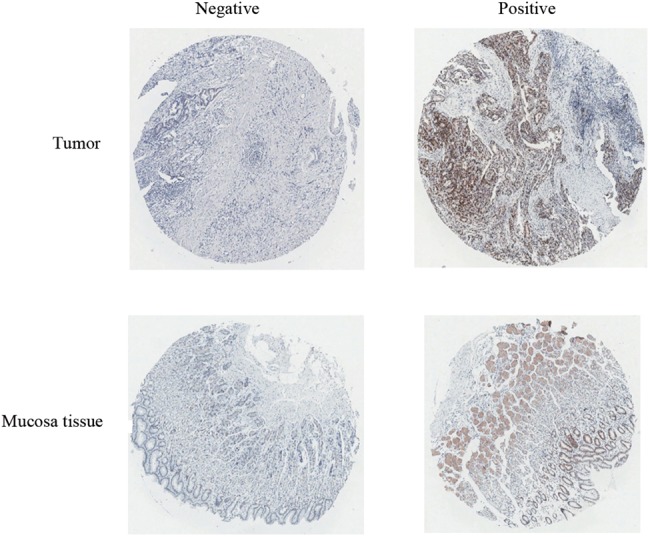
Ubqln4 expression in gastric tumor and normal gastric mucosa tissues. Representative images of Ubqln4 immunohistochemical staining in gastric tumor and normal gastric mucosa tissues. Negative and positive expression of Ubqln4 (from left to right) in gastric tumor and normal gastric mucosa tissues (top row and bottom row) are shown. Magnification ×20

We first examined the expression level of Ubqln4 in 94 pairs of GC and paired adjacent normal tissues using immunohistochemistry. Ubqln4 expression was evaluated and scored by two pathologists. Both tumor and normal mucosa tissues showed positive and negative staining of Ubqln4 (Fig. 1). To precisely evaluate Ubqln4 expression in GC tissues, we evaluated Ubqln4 expression as described in Methods [30]. The positive rates of Ubqln4 in normal gastric mucosa and GC tissues were 38.3% and 23.4%, respectively (Table 1). The Wilcoxon rank-sum test confirmed a statistically significant lower expression of Ubqln4 in GC tissues compared with normal tissues (*P* < 0.05). We also examined the relation between Ubqln4 expression level and the clinicopathological characteristics of patients from whom tissue samples were derived. However, no correlation between Ubqln4 expression level with lymph node invasion, distal metastasis, tumor size, or Tumor-Node-Metastasis (TNM) stage was observed (Supplementary Table S1).

**Table 1:** Expression level of Ubqln4 in gastric tissue microarray

Histologic classification		Ubqln4 expression level				No. of positive samples (%)	Statistical significance
	*n*	(−)	(+)	(++)	(+++)		
Adjacent gastric mucosa and cancer distal	94	58	21	13	2	38.3	*P* = 0.008*
Primary gastric cancer	94	72	16	5	1	23.4	

Wilcoxon rank-sum test

*Statistical significance

### Overexpression of Ubqln4 significantly suppresses GC cell growth in vivo and in vitro

**Fig. 2:**
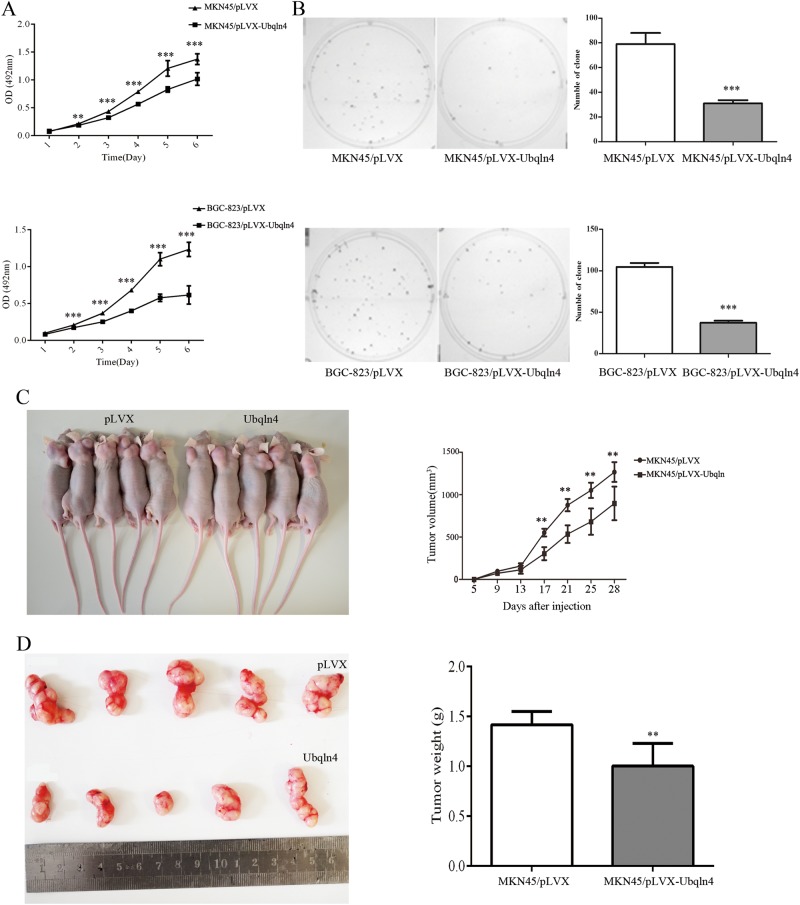
Ubqln4 represses GC cell proliferation in vitro and in vivo. Stable MKN45 and BGC-823 cell lines were generated by infection with Ubqln4 or control lentiviruses for 24 h and screening in 2 μg/ml puromycin for 2 weeks. **a** Indicated cell lines were plated into 96-well plates and cell viability was examined every 24 h by MTT assay. Each point represents mean ± SD of duplicates (*n* = 8). Student’s *t*-test; **P < 0.01, ***P < 0.001. **b** Colony-formation assay in MKN45 and BGC-823 stable cell lines expressing Ubqln4 compared with controls after 14 days of culture. Colony numbers in triplicates in three experiments were counted. Values are presented as mean ± SD. ***P < 0.001. **c** Xenograft tumors were established by injection of MKN45 cells stably expressing Ubqln4 compared with the controls (*n* = 5, group). Representative images of nude mice are shown. Tumor mass volume was every 4 days after injection. Measurements were repeated three times. Values are presented as mean ± SD. Student’s *t*-test. **P < 0.01. **d** Images of tumor xenografts from the indicated groups. Tumor weight was measured after mice was killed at the 28th day. Values are presented as mean ± SD. Student’s *t*-test. **P < 0.01

To investigate the role of Ubqln4 in GC, we generated Ubqln4 lentivirus and established stable Ubqln4-expressing cell lines in MKN45 and BGC-823 GC cells. Elevated Ubqln4 expression in the stable cell lines was confirmed by western blot (Supplementary Fig S1A). MTT and colony-formation assays showed that both MKN45 and BGC-823 stable cell lines overexpressing Ubqln4 exhibited significantly inhibited cell growth and colony-formation activities compared with control cells (Fig. 2a, b). The suppressive function of Ubqln4 on cell proliferation was further examined using tumor growth assays. Xenograft tumors from MKN45 stable cell lines overexpressing Ubqln4 had smaller mean tumor volume and weight compared with tumors from control cells (Fig. 2c, d). Together these data indicated that Ubqln4 functions as a tumor-inhibiting factor and negatively regulates gastric tumor growth.

### Ubqln4 inhibits G1-S transition of cell cycle and induces cellular senescence

**Fig. 3:**
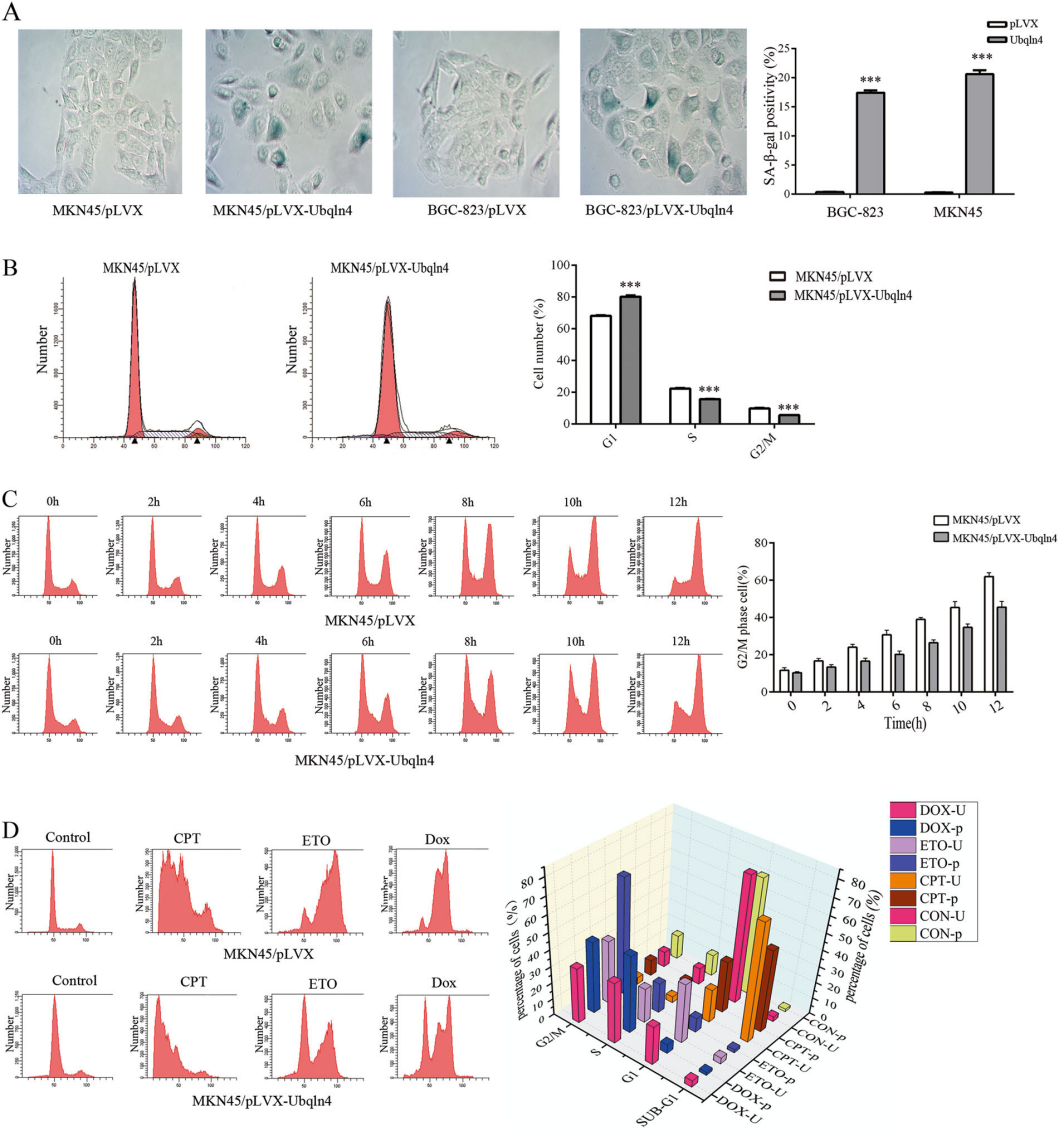
Ubqln4 induces cellular senescence and cell cycle arrest. **a** Induction of cellular senescence by Ubqln4 overexpression. Senescence-associated β-galactosidase (SA-β-Gal) staining was performed in MKN45 and BGC-823 stable cell lines overexpressing Ubqln4 and the respective controls. Data represents mean ± SD of triplicate assays. Student’s *t*-test, ***P < 0.001. **b** Cell cycle distributions of MKN45 stable cell line overexpressing Ubqln4 and the control cells were examined using flow cytometry. Percentages of cells in each phase are indicated. Student’s *t*-test, ***P < 0.001. **c** MKN45 stable cell lines overexpressing Ubqln4 and control cells were treated with nocodazole (100 μg/ml). Cell cycle distributions of cells harvested every 2 h for 12 h are shown. Quantification of G2/M cells are shown on the right. **d** MKN45 cells were treated with camptothecin (CPT, 1 μM), etoposide (ETO, 10 μM), or doxorubicin (DOX, 1 μM) for 24 h. Cell cycle distributions of cells were determined. On the right, the height of the bars represents the mean of the percentage of cells (‘p’, pLVX, or ‘U’, Ubqln4-overexpressing) in each phase. All experiments were performed in biological triplicates with three technical replicates

Interestingly, during cell culture of the MKN45 and BGC-823 stable cell lines overexpressing Ubqln4, we observed changes in cellular morphology; the cells became larger and flattened, which is a typical sign of cell senescence. We thus next performed senescence-associated β-galactosidase (SA-β-Gal) staining to evaluate senescent cells. The proportion of senescent cells in the Ubqln4-expressing MKN45 and BGC-823 cell lines was higher (17.4% ± 0.89% and 20.6% ± 1.52%, respectively) compared with the control cells (0.34% ± 0.09% and 0.28% ± 0.04%, respectively; *P* < 0.001) (Fig. 3a).

To elucidate the molecular mechanism by which Ubqln4 inhibits cell growth, we next investigated the effect of Ubqln4 on cell cycle distribution. GC stable cell lines overexpressing Ubqln4 exhibited an increase in G1 phase cells and a concomitant decrease in S phase cells and G2/M phase cells compared with the control cell lines (G0/G1: 80.11% ± 1.37% vs. 68.1% ± 0.4%, respectively; S: 15.59% ± 0.34% vs. 22.18 ± 0.83%; G2/M: 5.51% ± 0.16% vs. 9.81% ± 0.36%; *P* < 0.001) (Fig. 3b). To further clarify the influence of Ubqln4 on the cell cycle, we used nocodazole, a microtubule depolymerizing agent [30], to induce a G2/M phase arrest. We collected cells every 2 h and examined cell cycle distributions. Increased expression of Ubqln4 weakened the efficacy of nocodazole, which verified that Ubqln4-induced G1 phase arrest so that fewer cells could be arrested at G2/M phase by nocodazole (Fig. 3c). We also found that Ubqln4-overexpressing MKN45 cells displayed enhanced G1 arrest in response to various therapeutic agents, including camptothecin, etoposide and doxorubicin (Fig. 3d). Moreover, increased expression of Ubqln4 in GES-1, a human immortalized normal gastric epithelial cell line revealed cell shrinkage and aggregation, which reflect the morphological changes of cell apoptosis (Supplementary Fig S1B). Flow cytometry further showed a significantly higher percentage of Annexin V-positive cells in Ubqln4-overexpressing GES-1 cells compared with controls (13.1% ± 1.8% vs. 3.4% ± 1.1%) (Supplementary Fig S1C). Collectively, our data suggested that Ubqln4 expression-induced cell senescence, cell cycle arrest at G1 phase and apoptosis.

### Gene expression profiles in MKN45 cells with overexpression and knockdown of Ubqln4

To gain more insight into the mechanism underlying the effects of Ubqln4 on GC cells, we performed DNA microarray assays and screened the differentially expressed genes (DEGs) resulting from overexpression and knockdown of Ubqln4 in MKN45 cells. A gene set of 778 upregulated genes and 678 downregulated genes were differentially expressed by Ubqln4 overexpression at a threshold of >1.5-fold changes. We further analyzed these genes and found that 450 genes were upregulated in the Ubqln4 overexpression group and concurrently downregulated in the Ubqln4 knockdown group (Supplementary Table S2) and 424 genes were downregulated in the Ubqln4 overexpression group and upregulated in the Ubqln4 knockdown group (Supplementary Table S3).

DEGs were subjected to Gene Ontology (GO) analysis of biological processes, and we observed enrichment on items focusing on DNA repair, cellular response to DNA damage stimulus, DNA synthesis involved in DNA repair and cell cycle checkpoint (Supplementary Fig S2A). From the perspective of Molecular Function, DEGs mainly focused on protein homodimerization activity, single-stranded DNA binding and DNA-directed DNA polymerase activity (Supplementary Fig S2B). Cellular Component Analysis, another aspect of GO analysis, showed that DEGs were mostly enriched on lysosome membrane, nucleoplasm and extracellular exosome (Supplementary Fig S2C). We next performed pathway analysis and found several KEGG pathways involved, including the metabolic pathway, p53 signaling pathway, and pathways in cancer and lysosome (Supplementary Fig S2D). Consolidating the above results, we found a close connection between Ubqln4 and the p53 signaling pathway. There were 11 DEGs, *CDKN1A*, *CCNE2*, *FAS*, *PUMA*, *TP53I3*, *IGFBP3*, *PAI*, *SERPINB5*, *P53R2*, *SESN1*, and *MDM2* genes, in the p53 signaling pathway that were enriched.

**Fig. 4:**
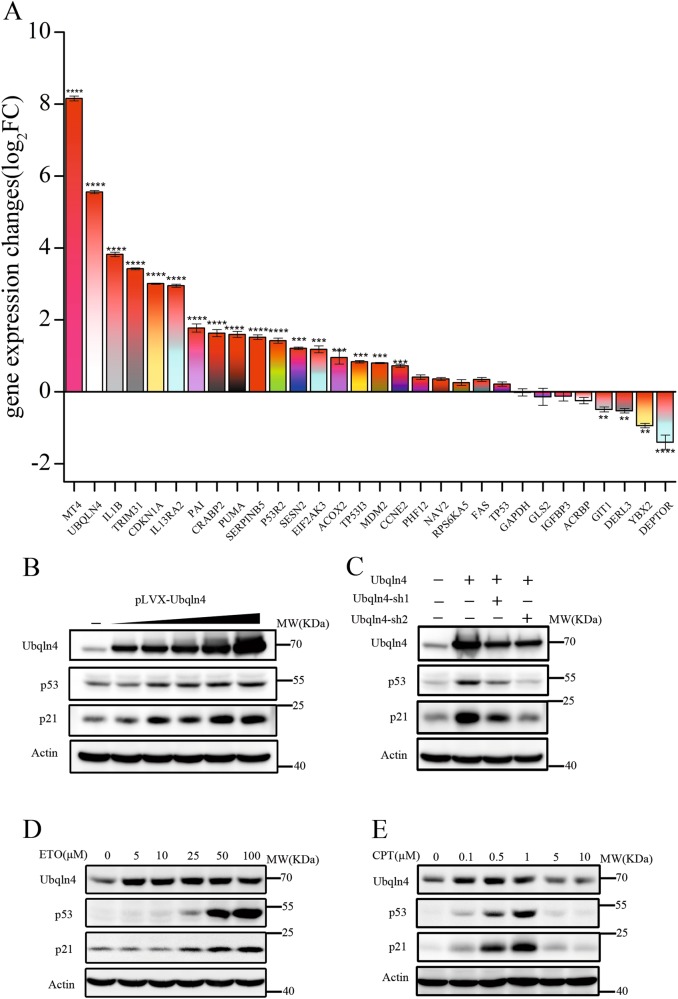
Ubqln4 induces alterations of the p53 signaling pathway in MKN45 cells. **a** Gene expression changes (log2FC) of 27 selected genes representing the p53 signaling pathway and DEGs in MKN45 cells with overexpression of Ubqln4 using quantitative RT-PCR. All quantitative RT-PCR experiments were performed in triplicate. Student’s *t*-test. **P < 0.01, ***P < 0.001, ****P < 0.0001. **b** Western blot analysis for the indicated proteins in MKN45 cells infected with Ubqln4 lentivirus at various concentrations (1:0.25, 1:0.5, 1:1, 1:2, and 1:4) for 48 h. **c** Western blot analysis for the indicated proteins in MKN45 cells infected with Ubqln4 lentivirus alone or together with Ubqln4-sh1 or Ubqln4-sh2 lentivirus as indicated. MKN45 cells infected with control lentivirus were used as controls. **d**, **e** MKN45 cells were treated with (**d**) etoposide (ETO) or (**e**) camptothecin (CPT) at the indicated concentrations for 24 h and Western blot was performed with the indicated antibodies

We next examined the expression of 27 selected genes representing the p53 signaling pathway and other pathways in Ubqln4-overexpressing MKN45 cells using quantitative RT-PCR. The gene expression changes of the representative DEGs in Ubqln4-overexpressing MKN45 cells verified the DNA microarray assay results (Fig. 4a, Supplementary Table S4) and highlighted the strong association between Ubqln4 and the p53 signaling pathway.

### Ubqln4 is a positive regulator of the p53/p21 axis

On the basis of the DNA microarray results, we next examined p53 expression. We found that p53 and p21 expression levels were markedly enhanced by Ubqln4 overexpression (Fig. 4b, c), and more importantly, the p53 and p21 protein levels increased in parallel with progressively increasing Ubqln4 expression, suggesting that Ubqln4 positively regulates p53 and p21 in a dose-dependent way (Fig. 4b). We constructed an Ubqln4 shRNA vector (Supplementary Fig S3A) and knocked down Ubqln4 in MKN45 Ubqln4-expressing stable cells; the expression level of p53 and p21 decreased as expected (Fig. 4c). We further induced p53 expression in MKN45 cells using etoposide, a topoisomerase II inhibitor that causes double strand DNA breaks [31], and found that the endogenous expression levels of p53, p21, and Ubqln4 were enhanced in MKN45 cells treated with etoposide in a dose-dependent manner (Fig. 4d). Notably, we observed an increase in Ubqln4 levels before p53 and p21 increases in MKN45 cells after etoposide treatment. To determine whether this was a drug-specific response, we performed similar experiments using camptothecin, a topoisomerase I inhibitor. In contrast to etoposide treatment, we observed decreased Ubqln4, p53, and p21 levels following an initial increase in cells treated with various concentrations of camptothecin (Fig. 4e). However, both results showed changes in Ubqln4 expression levels before p53 and p21 in cells suffering from DNA damage. These results suggest that during the DNA damage response, p53 and p21 may be regulated by Ubqln4.

### Ubqln4 stabilizes p21 by inhibiting its degradation in a p53-independent manner

**Fig. 5:**
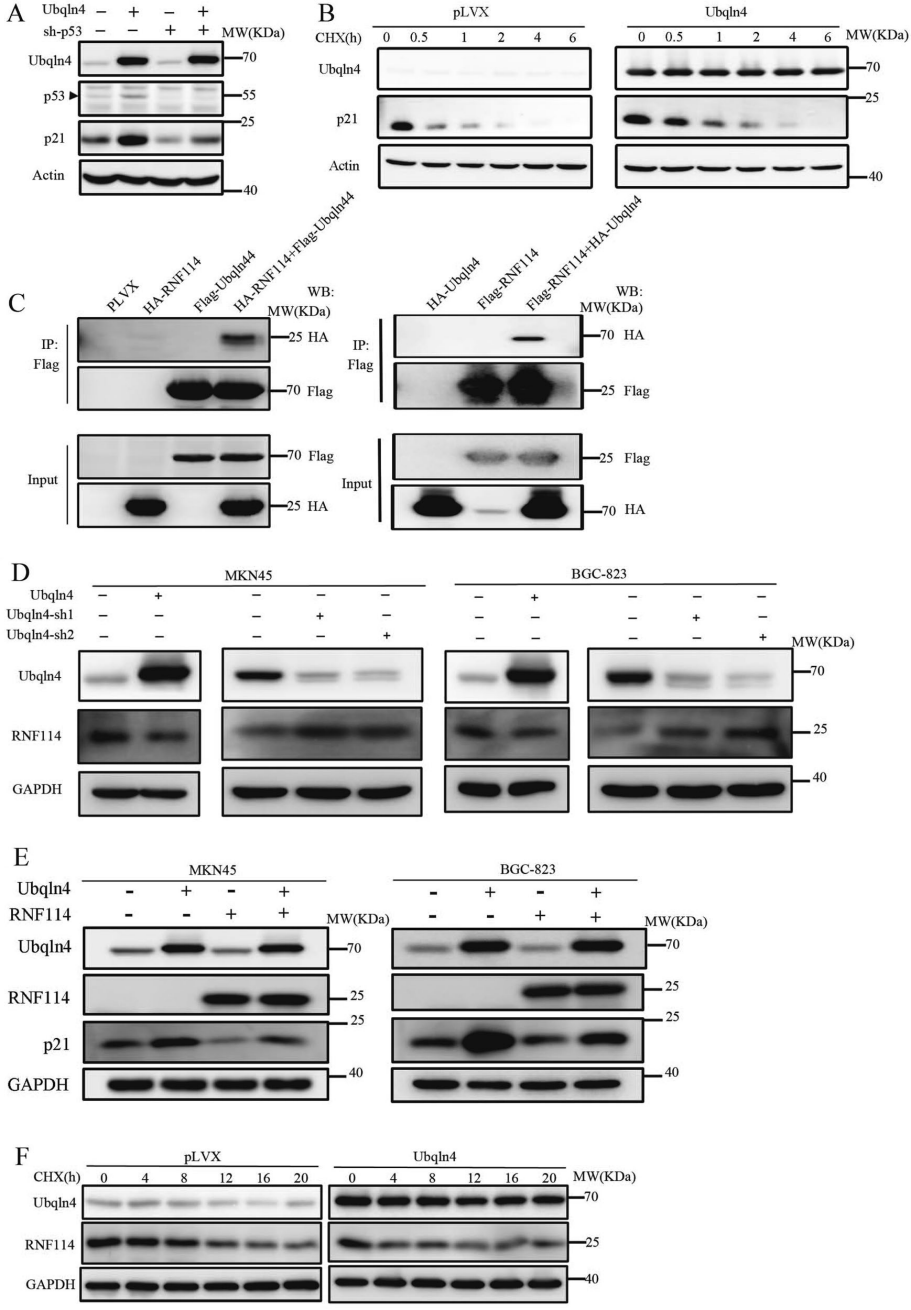
Ubqln4 inhibits p21 degradation via RNF114. **a** Western blot analysis in MKN45 cells infected with Ubqln4 lentivirus alone or with p53-sh lentivirus for 48 h has indicated. Control cells were infected with empty lentivirus. **b**, **f** Stable Ubqln4-expressing MKN45 cells and control MKN45 cells were treated with CHX (100 μg/ml) for **b** 0, 0.5, 1, 2, 4, or 6 h or **f** 0, 4, 8, 12, 16, or 20 h and cells were harvested and blotted against antibodies of **b** p21 or **f** RNF114. **c** 293T cells were transiently transfected with the indicated plasmids and Ubqln4 immunoprecipitation (left panel) or RNF114 immunoprecipitation (right panel) was performed using Flag antibodies. **d** Western blot analysis of MKN45 and BGC-823 cells infected with Ubqln4 lentivirus alone or with Ubqln4-sh1, Ubqln4-sh2, or GFP-sh lentivirus for 48 h. Cells infected with empty vector lentivirus served as controls. **e** Western blot analysis of MKN45 and BGC-823 cells infected with Ubqln4 lentivirus, RNF114 lentivirus or both Ubqln4 lentivirus and RNF114 lentivirus for 48 h. Cells infected with empty vector lentivirus served as controls

Our results showed that p53 and p21 expressions were influenced by Ubqln4. In addition to p53, p21 can also be regulated by other factors, such as TGFβ or mimosine in p53-independent manner [24]. We thus next evaluated whether Ubqln4 could regulate p21 independent of p53. We silenced p53 with or without overexpression of Ubqln4 in MKN45 cells. As expected, the expression level of p21 was decreased after p53 knockdown in MKN45 cells (Fig. 5a); however, overexpression of Ubqln4 in MKN45 cells with p53 knockdown still induced p21 levels. These data suggest that Ubqln4 regulates p21 levels through p53-dependent and p53-independent manners.

We speculated that Ubqln4 may regulate p21 levels through the ubiquitin-proteasome system. We thus used cycloheximide (CHX) to determine the half-life of endogenous p21 in MKN45 cells. The half-life of p21 was significantly prolonged by Ubqln4 overexpression in MKN45 cells (Fig. 5b), supporting the notion that Ubqln4 enhanced p21 protein stability by inhibiting its degradation. Overexpression of Ubqlns can cause the dysregulation of protein degradation relying on their interaction with the proteasome and ubiquitinated substrates. Overexpression of Ubqln4 may cause protein accumulation simply because of a slowing of cellular proteasomal degradation rates rather than itself physiological function. To address this issue, we found that there are some other proteins, c-Myc and EZH2, which actually do not accumulate upon Ubqln4 overexpression. That means the effect of Ubqln4 on p21 is specific rather than non-specific (Supplementary Fig S3B, C).

We next explored the molecular mechanism through which Ubqln4 inhibits p21 degradation by examining potential interacting proteins of Ubqln4. We isolated nuclear and cytoplasmic components and identified Ubqln4 interacting proteins by mass spectrometry (Supplementary Table S5). In mass spectrometry results, we also found some proteins known to bind to Ubqlns, such as Ubqln1 and BAG, which validated the mass spectrometry experiment [15, 32]. Among the identified proteins, RNF114, which possesses E3 ligase activity for p21, attracted our attention. We performed co-immunoprecipitation assays in 293T cells and confirmed that Ubqln4 could bind to RNF114 (Fig. 5c). Given our observation that Ubqln4 positively regulated p21 protein stability, we proposed that RNF114 would be the way through which p21 was affected by Ubqln4. In agreement with our hypothesis, we observed a negative correlation between Ubqln4 expression level and RNF114 levels in MKN45 and BGC-823 cells (Fig. 5d). We further found that overexpression of RNF114 downregulated p21 and that RNF114 overexpression partly blocked the induction of p21 resulting from Ubqln4 expression (Fig. 5e). The same results were found in BGC-823 cells. CHX assays demonstrated that Ubqln4 overexpression accelerated the rate of degradation of either endogenous or ectopic RNF114 protein (Fig. 5f and Supplementary Fig S3D). Together these data suggested that in addition to its p53-dependent effects, Ubqln4 can also regulate p21 by negatively regulating the E3 ligase RNF114.

### Effect of Ubqln4 on GC cells is mediated by p21

**Fig. 6:**
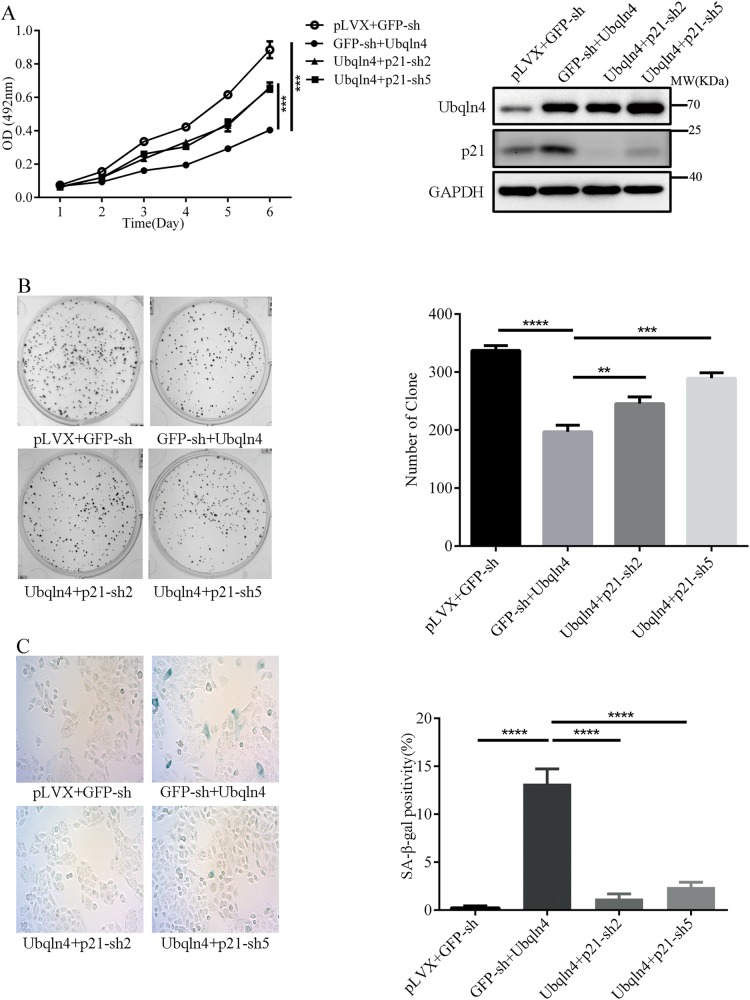
Effects of Ubqln4 in GC cells are mediated by p21. MKN45 cells were infected with Ubqln4 lentivirus alone or together with p21-sh2, p21-sh5, or GFP-sh lentivirus. Cells infected with empty vector lentivirus served as controls. Cells were screened in 2 μg/ml puromycin for 5 days. **a** MTT assay, **b** colony-formation assays, and **c** SA-β-Gal staining assays were conducted in the indicated cell lines as described in Figs. 2 and 3. Student’s *t*-test, **P < 0.01, ***P < 0.001

As a regulator of cell cycle and genomic stability, p21 is considered a tumor suppressor. On the basis of our results showing a tumor-suppressive influence of Ubqln4 on GC cells, we next evaluated whether the effects of Ubqln4 are mediated by p21. We constructed a lentivirus expressing p21 shRNA and confirmed effective knockdown of p21 (Supplementary Fig S3E, F). MTT assays showed that knockdown of p21 significantly, but not completely, abrogated the effect of Ubqln4 on GC cell viability (Fig. 6a) and colony-formation activities (Fig. 6b). Additionally, compared with overexpression of Ubqln4 alone, knockdown of p21 markedly decreased the proportion of senescent cells (13.8% ± 1.53% in GFP-sh + Ubqln4 cells vs. 1.24% ± 0.56% in Ubqln4 + p21-sh-2 or 2.4% ± 0.60% in Ubqln4+p21-sh5 cells) (Fig. 6c). These results confirmed that p21 is a mediator between Ubqln4 and its anti-oncogenic effects in GC cells.

## Discussion

Several studies have been reported on Ubqln4; however, little is known about the function of Ubqln4 in cancer, especially in GC. Our study is the first to provide experimental evidence for Ubqln4 downregulation in primary GC tissues. We further demonstrated that the cellular mechanism of Ubqln4 is focused on the p53/p21 axis and that Ubqln4 functions as a positive regulator of the p53/p21 axis.

The p53 pathway is a significant tumor suppressor pathway that leads to cell cycle arrest, apoptosis or cellular senescence [33, 34]. Here, we revealed the close connection between Ubqln4 and p53 from multiple aspects. First, analysis of the gene expression profiles in GC cells after overexpression or knockdown of Ubqln4 indicate, to a large extent, the intersection of Ubqln4 regulated genes with p53-related biological processes and molecular functions, including DNA repair, cellular response to DNA damage stimulus, cell cycle checkpoint, and single-strand DNA binding, among others. Among DEGs, we identified 11 genes, *CDKN1A*, *CCNE2*, *FAS*, *PUMA*, *TP53I3*, *IGFBP3*, *PAI*, *SERPINB5*, *P53R2*, *SESN1*, and *MDM2* genes, in the p53 signaling pathway that were enriched. We also detected an intensified G1 phase arrest response to DNA damage in Ubqln4-overexpressing GC cells treated with several well-known DNA damaging agents, including camptothecin, etoposide, and doxorubicin, compared with control cells. These observations indicate that elevated Ubqln4 may amplify and enhance the p53 response to damage in inducing cell cycle arrest in these cells. Third, we observed a faster induction of Ubqln4 levels to camptothecin and etoposide compared with p53, indicating that Ubqln4 may regulate endogenous p53 expression levels. We speculate that p53 may be a key factor in the tumor suppressor function of Ubqln4 in GC.

In response to DNA damage, p21 expression is elevated through the activity of the p53 transcription factor [35]. In this study, we found that Ubqln4 regulated p21 levels in both p53-dependent and p53-independent manners. Our results showed that Ubqln4 is a positive regulator of p53, which results in increased p21 levels. Intriguingly, we found that p53 knockdown could not completely eliminate the increase in p21 level induced by Ubqln4 overexpression. We thus hypothesized that p21 was additionally regulated by Ubqln4 in a p53-independent manner. Consistent with a previous report, we found that p21 is an unstable protein with a half-life of about 20–60 min [29]. However, ectopically expressed Ubqln4 markedly prolonged the half-life of p21. This indicates a strong role for Ubqln4 in regulating the stability of p21. We identified RNF114 (ZNF313) as an Ubqn4-interacting protein using immunoprecipitation and mass spectrometry, and verified its interaction with Ubqln4. RNF114 is a newly reported E3 ligase for p21, which interferes with p21-mediated cell cycle inhibition and cellular senescence by promotion of p21 degradation [25]. Our results showed that Ubqln4 negatively regulated RNF114 expression levels. Together these findings suggest that the cell cycle inhibitor p21 is a downstream molecule of Ubqln4 that is regulated by diverse mechanisms.

Cell proliferation is essential for tumorigenesis, and substantial evidence has demonstrated that cellular senescence is a mechanism that can restrain cancer cell proliferation [36]. Consistent with this notion, we observed that overexpression of Ubqln4 dramatically suppressed GC cell proliferation in vivo and in vitro and elicited senescence. Senescence is often initiated by various stimulus associated with DNA damage, and the p53/p21 pathway is a significant tumor suppressor pathway that regulates senescence [37]. Senescence can be observed in tumor cells only 2 or 3 days after p21 is overexpressed [38]. These findings suggest that Ubqln4-induced senescence could be the consequence of activation of the p53/p21 axis. One of the hallmarks of cellular senescence is disrupted cell cycle progression, and senescent cells typically show G1 phase arrest [36]. Induction of p21 by p53 upon DNA damage results in inhibited cyclin E/CDK2 and G1/S transition [20]. In this study, overexpression of Ubqln4 caused a G1 phase arrest, which supported a tumor suppressor function of Ubqln4. Moreover, knockdown of p21, to some extent, but not completely, abolished the effect of Ubqln4 on GC cells, illustrating that the effect of Ubqln4 is partly dependent on p21.

Interestingly, we also observed an induction of apoptosis in response to Ubqln4 overexpression in GES-1 cells. We speculated there may be some cell line-specific responses that contribute to the pro-apoptosis instead of senescence response. Activation of p53 tends to induce apoptosis in some types of cell lines, such as lymphocytes and transformed fibroblasts because of differences in the threshold for the onset of apoptosis in different cell types [39]. p53 also regulates the expression of many apoptosis-related genes, including *PUMA*, *NOXA*, *FAS*, and *APADF1* genes [40].

**Fig. 7:**
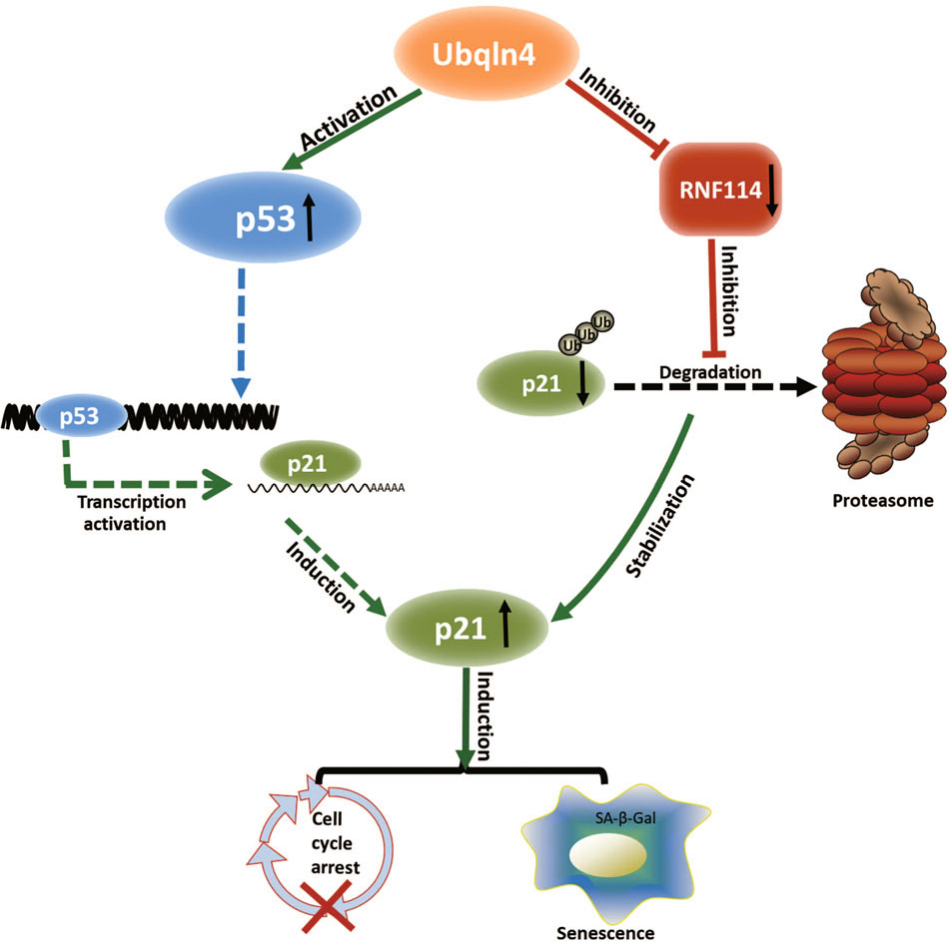
Ubqln4 functions as a tumor suppressor by regulating p21. Ubqln4 can activate p53, which results in induction of p21. In addition, Ubqln4 can also interact with and suppress RNF114, an E3 ligase of p21, which inhibits p21 degradation via the proteasomal pathway. The activation of p21 through different mechanistic pathways ultimately induces cell cycle arrest and/or cell senescence

The major finding of this study is that Ubqln4 acts as a tumor suppressor in the context of GC by inducing cell cycle arrest and senescence. Figure 7 presents the model of how Ubqln4 functions as a tumor suppressor. Overexpression of Ubqln4 on one hand activates the p53/p21 pathway, and on the other hand, it serves as a key regulator in modulating p21 stability via inhibiting ubiquitin ligase RNF114. Our data suggest that p53/p21 is at least partially responsible for the function of Ubqln4 in gastric tumorigenesis. However, we do not exclude other pathways of Ubqln4 in this sophisticated biological process.

## Materials and methods

### GC tissue microarray and immunohistochemistry

A 208-spot, paraffin-embedded, human GC tissue microarray (ST2084) containing 104 paired gastric tumor and adjacent gastric mucosa tissues was purchased from Alenabio (Xi’an, China). Immunohistochemical staining was performed as previously described [41]. On the basis of the previous report [42], the immunoreactivity of Ubqln4 was scored in a semiquantitative way by incorporating both stain intensity and the percentage of positive tumor cells. Ubqln4 intensity was scored as 0 (no staining), 1 (weak staining), 2 (moderate staining), or 3 (strong staining). The percentage of positive cells was scored as 0 (no positive cells), 1 (<10%), 2 (10–50%), or 3 (>50%). The final score was obtained by multiplying the score of intensity by the score of the percentage of positive cells. Cases with a score of ≤1 were considered negative (−). Cases with higher scores were considered positive and further classified as follows: weak positive cases (+) with a final score of 2–3, moderate positive cases (++) with a final score of 4–6 and strong positive cases (+++) with a final score of 7–9. Sections were evaluated independently by two pathologists and a final agreement was acquired for each score. Among total samples, 20 samples were excluded because of gastritis diagnosed by pathologists.

### Cell culture and plasmid construction

GC cell lines MKN45 and BGC-823, human immortalized normal gastric epithelial GES-1 cells, human embryonic kidney epithelial 293T cells, and human cervical cancer HeLa cells were purchased from the Shanghai Institute of Biochemistry and Cell Biology, Chinese Academy of Sciences (Shanghai, China) and maintained in Dulbecco’s Modified Eagle Media (HyClone, Utah, USA) supplemented with 10% heat-inactivated fetal bovine serum (FBS), 100 IU/ml penicillin and streptomycin at 37 °C in a humidified atmosphere containing 5% CO_2_. The Ubqln4/RNF114 cDNA was kindly provided by Dr. Jiahuai Han and was cloned into the pLVX-Puro vector with an N-terminal flag/HA tag. Four shRNA sequences for Ubqln4 and negative control shRNA were cloned into pSIH1-H1-Puro lentiviral vectors. The Ubqln4, p21, and p53 shRNA sequences were as follows:

Ubqln4-shRNA1: 5′-GGTCAGGGATGTTCAATAG-3′

Ubqln4-shRNA2: 5′-CAATAACCCTGAACTCAT G-3′

Ubqln4-shRNA3: 5′-CGACTTTGCTGCTCAGATG-3′

Ubqln4-shRNA4: 5′-GAGCCTCGGTCAAGGAGTT-3′

p21-shRNA1: 5′-AGCGATGGAACTTCGACTTT-3′

p21-shRNA2: 5′-GTCTCAGTTTGTGTGTCTTAAT-3′

p21-shRNA3: 5′-AGCCGCGACTGTGATGCGCTAA-3′

p21-shRNA4: 5′-GGCTGATCTTCTCCAAGAGGAA-3′

p21-shRNA5: 5′-CTGTCACAGGCGGTTATGAAAT-3′

p53-shRNA: 5′-GACTCCAGTGGTAATCTAC-3′

GFP-shRNA: 5′-CGAGAAGCGCGATCACATG-3′

### Lentivirus production and stable cell lines

Lentiviruses were produced by co-transfection of 293T cells with the lentiviral vector pLVX-Puro-containing Ubqln4/RNF14/Ubqln4-sh/p21-sh/p53-sh, and packaging vectors psPAX2 and pMD2.G with X-tremeGENE HP DNA transfection reagent (Roche, Switzerland) according to the manufacturer’s instructions. At 48 h post-transfection, lentiviral supernatant was collected and filtered. To establish stable cell lines, GC cells were infected with lentiviral supernatant for 24 h and then maintained with fresh complete medium containing 2 μg/ml puromycin for a continuous two-week period.

### MTT assay and colony-formation assay

Cells were plated into 96-well plates at a density of 1500 cells per well for MTT (*n* = 8) or six-well plates with 200–800 cells/well for colony-formation assay. For MTT, cell viability was examined every 24 h after seeding. Briefly, cells were incubated with 20 μl of MTT solution (5 mg/ml, Sigma, USA) combined with 90 μl DMEM at 37 °C for 4 h. The medium was aspirated and 150 μl of dimethyl sulphoxide (DMSO) was added to each well and absorbance at 492 nm was measured on a microplate spectrophotometer. All MTT assays were repeated three times. For colony formation, cells were cultured with complete medium for 10–14 days. Then, cells were fixed with 4% paraformaldehyde and stained with Giemsa solution. Visible colonies were counted. Each well was assessed in triplicate.

### In vivo tumorigenesis

BALB/c male nude mice were obtained from Beijing HFK Bioscience (Beijing, China). Cell suspensions (1 × 10^6^ cells in 0.1 ml PBS) were subcutaneously injected into the dorsal right flank of mice (*n* = 5 per group) aged 4 weeks of age. The longest and shortest diameters of tumor masses were measured every 4 days for 28 days and then mice were killed. Tumor volume was calculated as follows: volume (mm^3^) = (shortest diameter)^2^ × (longest diameter) × 0.5 [43]. After tumor excision, tumor weight was recorded. Care of animals and all animal-related experiments were performed according to the institutional and national guidelines for animal experiments.

### Senescence-associated β-galactosidase activity assay

Cells were seeded in 24-well plates at a density of 5000 cells/well 1 day before analyses. β-galactosidase activity was determined using a senescence cell histochemical staining kit (Cat. CS0030, Sigma-Aldrich, USA) according to the manufacturer’s recommendations. Senescent cells were counted under the microscope. Each sample was assayed in triplicate.

### Flow cytometric analysis

For cell cycle analysis, cells were harvested, washed with PBS and fixed in 75% ethanol at −20 °C overnight. RNA was removed by incubating the cells with RNase A (100 μg/ml, Sigma-Aldrich) and 0.25% (v/v) Triton X-100 at 37 °C for 30 min. Cells were then stained with propidium iodide (PI) solution (50 μg/ml, Sigma-Aldrich) for 30 min at RT and analyzed on flow cytometer. Cell apoptosis was assessed using a FITC Annexin V Apoptosis Detection Kit I (BD Pharmingen, San Diego, CA, USA) according to the manufacturer’s instructions.

### Gene expression profile and quantitative PCR (qPCR) analysis

The Agilent SurePrint G3Human GE 8*60K Microarray (G4851B) that covers 26, 083 Entrez Genes (unique) and 30, 606 long non-coding RNAs (unique) was used to compare DEGs between MKN45-control and Ubqln4-overexpressing MKN45 cells. GO analysis was performed using the Database for Annotation, Visualization, and Integrated Discovery 6.7 (DAVID6.7, http://david.abcc.ncifcrf.gov/home.jsp). We chose the DAVID default set of whole genome background of Homo sapiens for the DAVID analysis. The KEGG pathway analysis was conducted using KOBAS3.0 (http://kobas.cbi.pku.edu.cn/) [44]. Total RNA was extracted from cells using TRIzol reagent (Invitrogen, USA). cDNA synthesis was conducted using the PrimeScript™ 1st Strand cDNA Synthesis Kit (Takara, Japan). qPCR was performed using SYBR® Premix Ex Taq™ II (Tli RNaseH Plus, Takara) and the ABI ViiA™ 7 Fast Real-Time PCR System. Primers used for qPCR procedure are listed in Supplementary Table S4. mRNA expression of target genes was normalized to GAPDH and calculated by the 2^−ΔΔCT^ method [45]. All qPCR experiments were performed in triplicate. Primers used in this study were listed in Supplementary Table S4.

### Protein extraction and western blot

Protein extraction and western blot were performed as previously described [41, 46]. Antibodies used are as follows: Ubqln4 (A333, 1:1000, Santa Cruz, CA, USA); p53 (DO-1, 1:1000, Abcam, Cambridge, UK); p21 (EPR362, 1:1000, Abcam); RNF114 (SM-3, 1:1000, Santa Cruz); Flag (3B9, 1:5000, Abmart, Shanghai, China); HA (C29F4, 1:1000, CST); GAPDH (D16H11, 1:3000, CST); and β-actin (8H10D10, 1:1000, CST).

### Mass spectrometry

Flag-Ubqln4 HeLa cells were generated by lentivirus infection followed by puromycin selection as described above. Flag-Ubqln4 HeLa cells were cultured in 15 150-mm dishes (~3 × 10^8^ cells per plate). Cells were rinsed three times with ice-cold PBS and scraped into a microfuge tube. Cells were resuspended in 5 × PCV (packed cell volume) of ice-cold Buffer A (10 mM HEPES pH 7.9, 1.5 mM MgCl_2_, 10 mM KCl, 1 mM DTT, 0.5 mM PMSF, 1 × protease inhibitor cocktail). After incubation on ice for 10 min, the cells were harvested by centrifugation at 1000×*g*, 4 °C for 10 min. The cells were resuspended in 2 × PCV of ice-cold Buffer F1 (10 mM HEPES pH 7.9, 1.5 mM MgCl_2_, 10 mM KCl, 0.03% NP-40, 1 mM DTT, 0.5 mM PMSF, 1 × protease inhibitor cocktail) for 10 min to break the cell membrane. The mixture was then centrifuged at 1000×*g*, 4 °C for 10 min and then 5 M NaCl and 10% NP-40 were added to the supernatant to a final concentration of 300 mM NaCl and 0.5% NP-40. This sample was recorded as Fraction 1. The cell pellets were then extracted with 3.5 × PCV of ice-cold Buffer F2 (10 mM HEPES pH 7.9, 50 mM NaCl, 1.5 mM MgCl_2_, 10 mM KCl, 0.03% NP-40, 1 mM DTT, 0.5 mM PMSF, 1 × protease inhibitor cocktail) for 5 min to further extract cytosolic proteins. Mixtures were centrifuged at 1500×*g*, 4 °C for 10 min and then 5 M NaCl and 10% NP-40 were added to the supernatant to final concentrations of 300 mM NaCl and 0.5% NP-40. This sample was recorded as Fraction 2. To extract remaining proteins, the pellet was then extracted with 3.8 × PCV of ice-cold Buffer F3 (10 mM HEPES pH 7.9, 300 mM NaCl, 1.5 mM MgCl2, 10 mM KCl, 0.5% NP-40, 1 mM DTT, 0.5 mM PMSF, 1 × protease inhibitor cocktail) for 30 min. The final extraction was saved as Fraction 3. To purify Flag-Ubqln4-associated proteins from the prepared fractions, 30 μL anti-Flag M2 agarose beads were incubated with each fraction on a rotor at 4 °C for 8 h. Mixtures were washed extensively with Buffer F3 and then washed three times with PBS. Proteins on beads were directly denatured with 8 M Urea Buffer (PBS with 8 M Urea) followed by mass spectrometry analysis. Prior to trypsinization, samples were diluted to final 1.33 M of Urea with 50 mM NH_4_HCO_3_. Trypsin was added at a protein:trypsin ratio of 100:1 at 37 °C overnight. Peptides were then acidified by adding formic acid to a final concentration of 1% (vol/vol), pH 2–3, followed by loading onto balanced C18 STAGETIP. For desalting, peptides were washed twice with 1% formic acid and then eluted twice with 70% acetonitrile, 1% formic acid and then freeze-dried. The peptides were then dissolved in 2% acetonitrile, 0.1% formic acid for chromatography separation in an Eksigent NanoLC Ultra 2D Plus HPLC system (Eksigent, Dublin, CA, USA). Peptides were loaded onto a 75 µm × 150 mm fused silica microcapillary emitter (New Objective, Woburn, MA, USA) packed with 3 µm C18 resin (New Objective). Peptides were separated in a 60 min acetonitrile gradient from 2–35% (buffer A 0.1% formic acid, 2% acetonitrile; buffer B 0.1% formic acid, 98% acetonitrile). Peptides were then ionized in the positive ion mode by electrospray ionization (spray voltage = 2.2 kv) in a Nanospray III source (AB SCIEX, Concord, ON, CA, USA) and sprayed into a TripleTOF 5600 plus System (AB SCIEX, Foster City, CA, USA), a hybrid quadrupole TOF mass spectrum. For each sample, triple injections were applied. Tandem mass spectra were interpreted using ProteinPilot V4.5 beta against the human Uniprot proteome database appended with common contaminants and reversed-sequence decoys. Peptides and proteins were filtered with confidence corresponding to the 1% FDR. Protein summaries of each fraction were obtained from the Protein summary file exported from ProteinPilot V4.5 beta.

### Co-immunoprecipitation

For Ubqln4 and RNF114 co-immunoprecipitation experiments, 293T cells were transiently co-transfected with pLVX-Flag-Ubqnl4 and pLVX-HA-RNF114. Cells were harvested, washed with cold PBS, and lysed in a lysis buffer CF0.3 (30 mmol/l Hepes pH 7.9, 20% glycerol, 300 mmol/l NaCl, 0.5% NP-40, 0.4 mmol/l EDTA) supplemented with 0.5 mmol/l PMSF and 1 mmol/l DTT at 48 h after co-transfection. Immunoprecipitation was performed with mouse anti-Flag M2 affinity gel (Sigma-Aldrich). Proteins were further analyzed by western blotting or mass spectrometry.

### Statistical analysis

All statistical analyses were performed using IBM SPSS STATISTICS V20_32bit software (SPSS Inc., Chicago, IL, USA). Data were displayed as mean ± SD. The Wilcoxon rank-sum test was applied to analyze discontinuous variables. Comparison between multiple groups was conducted using Kruskal–Wallis test. Student’s *t*-test was used for statistical comparison between two groups. *P*-values < 0.05 indicated statistical significance.

## Electronic supplementary material

Supplementary Table S1

Supplementary Figure S1

Supplementary Figure S2

Supplementary Figure S3

Supplementary Table S2

Supplementary Table S3

Supplementary Table S4

Supplementary Table S5

Supplementary Figure Legends

## References

[1] Wadhwa R, Song S, Lee JS, Yao Y, Wei Q, Ajani JA (2013). Gastric cancer-molecular and clinical dimensions. Nat Rev Clin Oncol.

[2] Wagner AD, Unverzagt S, Grothe W, Kleber G, Grothey A, Haerting J, et al. Chemotherapy for advanced gastric cancer. Cochrane Database Syst Rev. 2010;3:CD004064. 10.1002/14651858.CD004064.pub3 20238327

[3] Whiteley AM, Prado MA, Peng I, Abbas AR, Haley B, Paulo JA (2017). Ubiquilin1 promotes antigen-receptor mediated proliferation by eliminating mislocalized mitochondrial proteins. eLife.

[4] Kleijnen MF, Alarcon RM, Howley PM (2003). The ubiquitin-associated domain of hPLIC-2 interacts with the proteasome. Mol Biol Cell.

[5] Walters KJ, Kleijnen MF, Goh AM, Wagner G, Howley PM (2002). Structural studies of the interaction between ubiquitin family proteins and proteasome subunit S5a. Biochemistry.

[6] Schauber C, Chen L, Tongaonkar P, Vega I, Lambertson D, Potts W (1998). Rad23 links DNA repair to the ubiquitin/proteasome pathway. Nature.

[7] Su V, Lau AF (2009). Ubiquitin-like and ubiquitin-associated domain proteins: significance in proteasomal degradation. Cell Mol Life Sci.

[8] Itakura E, Zavodszky E, Shao S, Wohlever ML, Keenan RJ, Hegde RS (2016). Ubiquilins chaperone and triage mitochondrial membrane proteins for degradation. Mol Cell.

[9] Kleijnen MF, Shih AH, Zhou P, Kumar S, Soccio RE, Kedersha NL (2000). The hPLIC proteins may provide a link between the ubiquitination machinery and the proteasome. Mol Cell.

[10] Hjerpe R, Bett JS, Keuss MJ, Solovyova A, McWilliams TG, Johnson C (2016). UBQLN2 mediates autophagy-independent protein aggregate clearance by the proteasome. Cell.

[11] Beverly LJ, Lockwood WW, Shah PP, Erdjument-Bromage H, Varmus H (2012). Ubiquitination, localization, and stability of an anti-apoptotic BCL2-like protein, BCL2L10/BCLb, are regulated by Ubiquilin1. Proc Natl Acad Sci USA.

[12] Shah PP, Lockwood WW, Saurabh K, Kurlawala Z, Shannon SP, Waigel S (2015). Ubiquilin1 represses migration and epithelial-to-mesenchymal transition of human non-small cell lung cancer cells. Oncogene.

[13] Wang Y, Lu J, Zhao X, Feng Y, Lv S, Mu Y (2015). Prognostic significance of Ubiquilin1 expression in invasive breast cancer. Cancer Biomark.

[14] Li X, Su V, Kurata WE, Jin C, Lau AF (2008). A novel connexin43-interacting protein, CIP75, which belongs to the UbL-UBA protein family, regulates the turnover of connexin43. J Biol Chem.

[15] Lee DY, Arnott D, Brown EJ (2013). Ubiquilin4 is an adaptor protein that recruits Ubiquilin1 to the autophagy machinery. EMBO Rep.

[16] Naus CC, Laird DW (2010). Implications and challenges of connexin connections to cancer. Nat Rev Cancer.

[17] van Veen AA, van Rijen HV, Opthof T (2001). Cardiac gap junction channels: modulation of expression and channel properties. Cardiovasc Res.

[18] Davidson JD, Riley B, Burright EN, Duvick LA, Zoghbi HY, Orr HT (2000). Identification and characterization of an ataxin-1-interacting protein: A1Up, a ubiquitin-like nuclear protein. Hum Mol Genet.

[19] Gartel AL (2006). Is p21 an oncogene?. Mol Cancer Ther.

[20] Bertoli C, Skotheim JM, de Bruin RA (2013). Control of cell cycle transcription during G1 and S phases. Nat Rev Mol Cell Biol.

[21] Jung YS, Qian Y, Chen X (2010). Examination of the expanding pathways for the regulation of p21 expression and activity. Cell Signal.

[22] Chang LJ, Eastman A (2012). Decreased translation of p21waf1 mRNA causes attenuated p53 signaling in some p53 wild-type tumors. Cell Cycle.

[23] Holland TA, Elder J, McCloud JM, Hall C, Deakin M, Fryer AA (2001). Subcellular localisation of cyclin D1 protein in colorectal tumours is associated with p21(WAF1/CIP1) expression and correlates with patient survival. Int J Cancer.

[24] Karimian A, Ahmadi Y, Yousefi B (2016). Multiple functions of p21 in cell cycle, apoptosis and transcriptional regulation after DNA damage. DNA Repair.

[25] Han J, Kim YL, Lee KW, Her NG, Ha TK, Yoon S (2013). ZNF313 is a novel cell cycle activator with an E3 ligase activity inhibiting cellular senescence by destabilizingp21(WAF1.). Cell Death Differ.

[26] Nakayama KI, Nakayama K (2005). Regulation of the cell cycle by SCF-type ubiquitin ligases. Semin Cell Dev Biol.

[27] Lee EW, Lee MS, Camus S, Ghim J, Yang MR, Oh W (2009). Differential regulation of p53 and p21 by MKRN1 E3 ligase controls cell cycle arrest and apoptosis. EMBO J.

[28] Xu S, Feng Z, Zhang M, Wu Y, Sang Y, Xu H (2011). hSSB1 binds and protects p21 from ubiquitin-mediated degradation and positively correlates with p21 in human hepatocellular carcinomas. Oncogene.

[29] Jascur T, Brickner H, Salles-Passador I, Barbier V, El Khissiin A, Smith B (2005). Regulation of p21(WAF1/CIP1) stability by WISp39, a Hsp90 binding TPR protein. Mol Cell.

[30] Signoretto E, Honisch S, Briglia M, Faggio C, Castagna M, Lang F (2016). Nocodazole induced suicidal death of human erythrocytes. Cell Physiol Biochem.

[31] Fitzgibbon C, Ihmaid S, Al-Rawi J, Meehan-Andrews T, Bradley C (2013). Chemo-sensitisation of HeLa cells to etoposide by a benzoxazine in the absence of DNA-PK inhibition. Invest New Drugs.

[32] Suzuki R, Kawahara H (2016). UBQLN4 recognizes mislocalized transmembrane domain proteins and targets these to proteasomal degradation. EMBO Rep.

[33] Giaccia AJ, Kastan MB (1998). The complexity of p53 modulation: emerging patterns from divergent signals. Genes Dev.

[34] Hu W, Feng Z, Levine AJ (2012). The regulation of multiple p53 stress responses is mediated through MDM2. Genes Cancer.

[35] Zhang L, Mei Y, Fu NY, Guan L, Xie W, Liu HH (2012). TRIM39 regulates cell cycle progression and DNA damage responses via stabilizing p21. Proc Natl Acad Sci USA.

[36] Campisi J, d’Adda, di Fagagna F (2007). Cellular senescence: when bad things happen to good cells. Nat Rev Mol Cell Biol.

[37] Jia L, Soengas MS, Sun Y (2009). ROC1/RBX1 E3 ubiquitin ligase silencing suppresses tumor cell growth via sequential induction of G2-M arrest, apoptosis, and senescence. Cancer Res.

[38] Chang BD, Xuan Y, Broude EV, Zhu H, Schott B, Fang J (1999). Role of p53 and p21waf1/cip1 in senescence-like terminal proliferation arrest induced in human tumor cells by chemotherapeutic drugs. Oncogene.

[39] Meek DW (2004). The p53 response to DNA damage. DNA Repair.

[40] Riley T, Sontag E, Chen P, Levine A (2008). Transcriptional control of human p53-regulated genes. Nat Rev Mol Cell Biol.

[41] Ma Y, Zhao M, Zhong J, Shi L, Luo Q, Liu J (2010). Proteomic profiling of proteins associated with lymph node metastasis in colorectal cancer. J Cell Biochem.

[42] Kusinska RU, Kordek R, Pluciennik E, Bednarek AK, Piekarski JH, Potemski P (2009). Does vimentin help to delineate the so-called ‘basal type breast cancer’?. J Exp Clin Cancer Res.

[43] Yu J, Cheng YY, Tao Q, Cheung KF, Lam CN, Geng H (2009). Methylation of protocadherin 10, a novel tumor suppressor, is associated with poor prognosis in patients with gastric cancer. Gastroenterology.

[44] Xie C, Mao X, Huang J, Ding Y, Wu J, Dong S (2011). KOBAS 2.0: a web server for annotation and identification of enriched pathways and diseases. Nucleic Acids Res.

[45] Livak KJ, Schmittgen TD (2001). Analysis of relative gene expression data using real-time quantitative PCR and the 2(-Delta Delta C(T)) Method. Methods.

[46] Zhong J, Zhao M, Ma Y, Luo Q, Liu J, Wang J (2012). UCHL1 acts as a colorectal cancer oncogene via activation of the beta-catenin/TCF pathway through its deubiquitinating activity. Int J Mol Med.

