# Supplementary material for: The UbL-UBA Ubiquilin4 protein functions as a tumor suppressor in gastric cancer by p53-dependent and p53-independent regulation of p21
Source: Cell Death Differ. 2018 Jun 13;26(3):516–30. doi: 10.1038/s41418-018-0141-4 (PMC6370890; doi:10.1038/s41418-018-0141-4)
Supplement: Supplementary file 1 — Supplementary Table S1 [file 41418_2018_141_MOESM1_ESM.docx]

Supplementary Table S1 Association of Ubqln4 expression with the clinicopathological features of patients with gastric cancer

| Clinicopathologic variables | n | Ubqln4 expression level | | | | Statistical significance |
| --- | --- | --- | --- | --- | --- | --- |
|  |  | （-） | （+） | （++） | （+++） |  |
| Gender |  |  |  |  |  | P=0.375 |
| male | 70 | 55 | 12 | 2 | 1 |  |
| female | 24 | 17 | 4 | 3 | 0 |  |
| Age |  |  |  |  |  | P=.922 |
| >=60 | 50 | 38 | 9 | 3 | 0 |  |
| <60 | 44 | 34 | 7 | 2 | 1 |  |
| TNM stage |  |  |  |  |  | P=0.160 |
| Ⅰ | 20 | 14 | 5 | 1 | 0 |  |
| Ⅱ | 48 | 35 | 8 | 4 | 1 |  |
| Ⅲ | 25 | 23 | 2 | 0 | 0 |  |
| Ⅳ | 1 | 1 | 0 | 0 | 0 |  |
| Primary tumor (T) stage |  |  |  |  |  | P=0.469 |
| T1 | 2 | 1 | 1 | 0 | 0 |  |
| T2 | 24 | 16 | 6 | 1 | 1 |  |
| T3 | 63 | 51 | 8 | 4 | 0 |  |
| T4 | 5 | 4 | 1 | 0 | 0 |  |
| Primary tumor (N) stage |  |  |  |  |  | P=0.374 |
| N0 | 66 | 48 | 13 | 5 | 0 |  |
| N1 | 24 | 21 | 2 | 0 | 1 |  |
| N2 | 4 | 3 | 1 | 0 | 0 |  |
| Primary tumor (M) stage |  |  |  |  |  | P=0.583 |
| M0 | 93 | 71 | 16 | 5 | 1 |  |
| M1 | 1 | 1 | 0 | 0 | 0 |  |

Kruskal-Wallis test
