# Supplementary figures and images for: The UbL-UBA Ubiquilin4 protein functions as a tumor suppressor in gastric cancer by p53-dependent and p53-independent regulation of p21
Source: Cell Death Differ. 2018 Jun 13;26(3):516–30. doi: 10.1038/s41418-018-0141-4 (PMC6370890; doi:10.1038/s41418-018-0141-4)

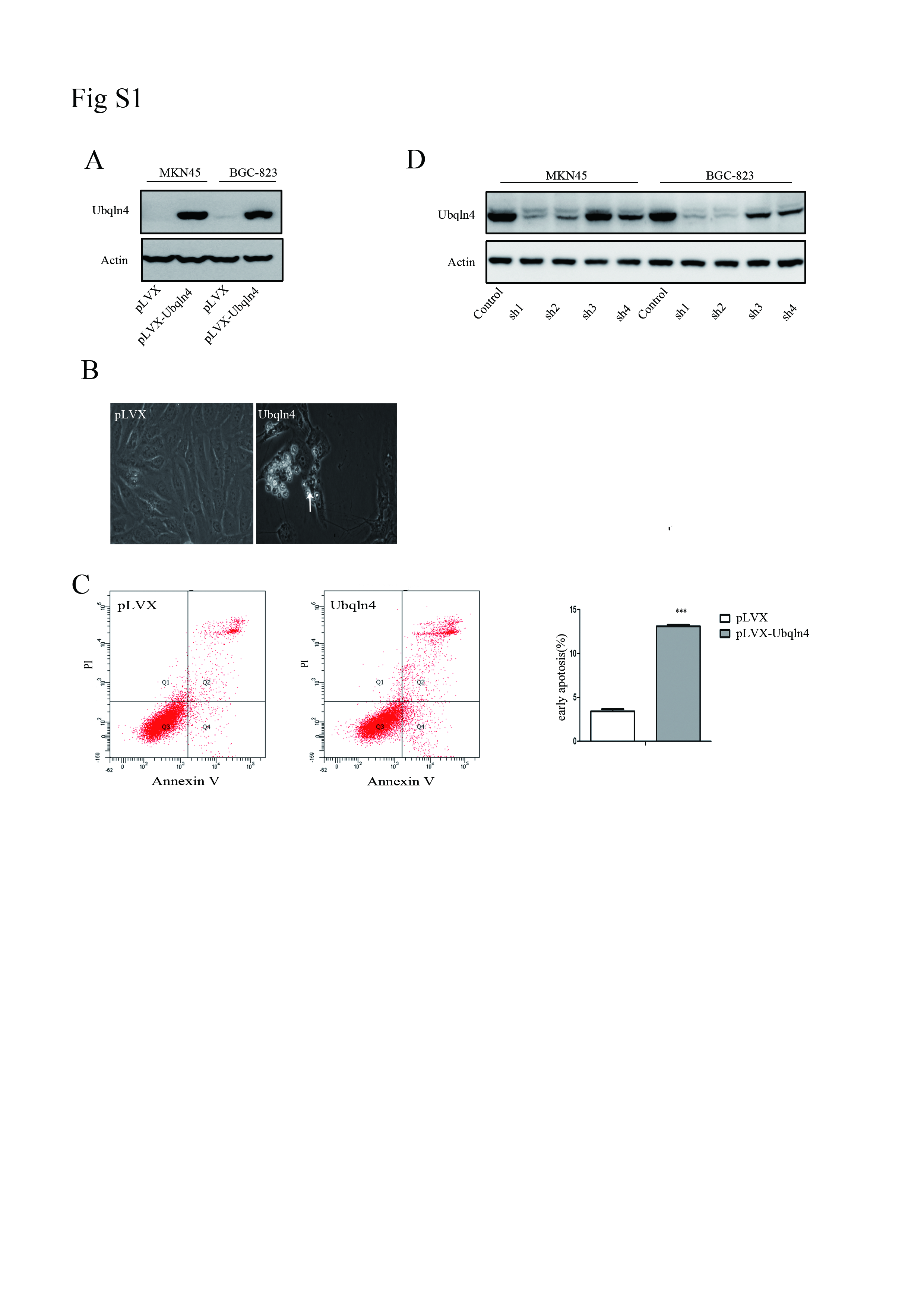

Supplement: Supplementary file 2 — Supplementary Figure S1 [file 41418_2018_141_MOESM2_ESM.tif]

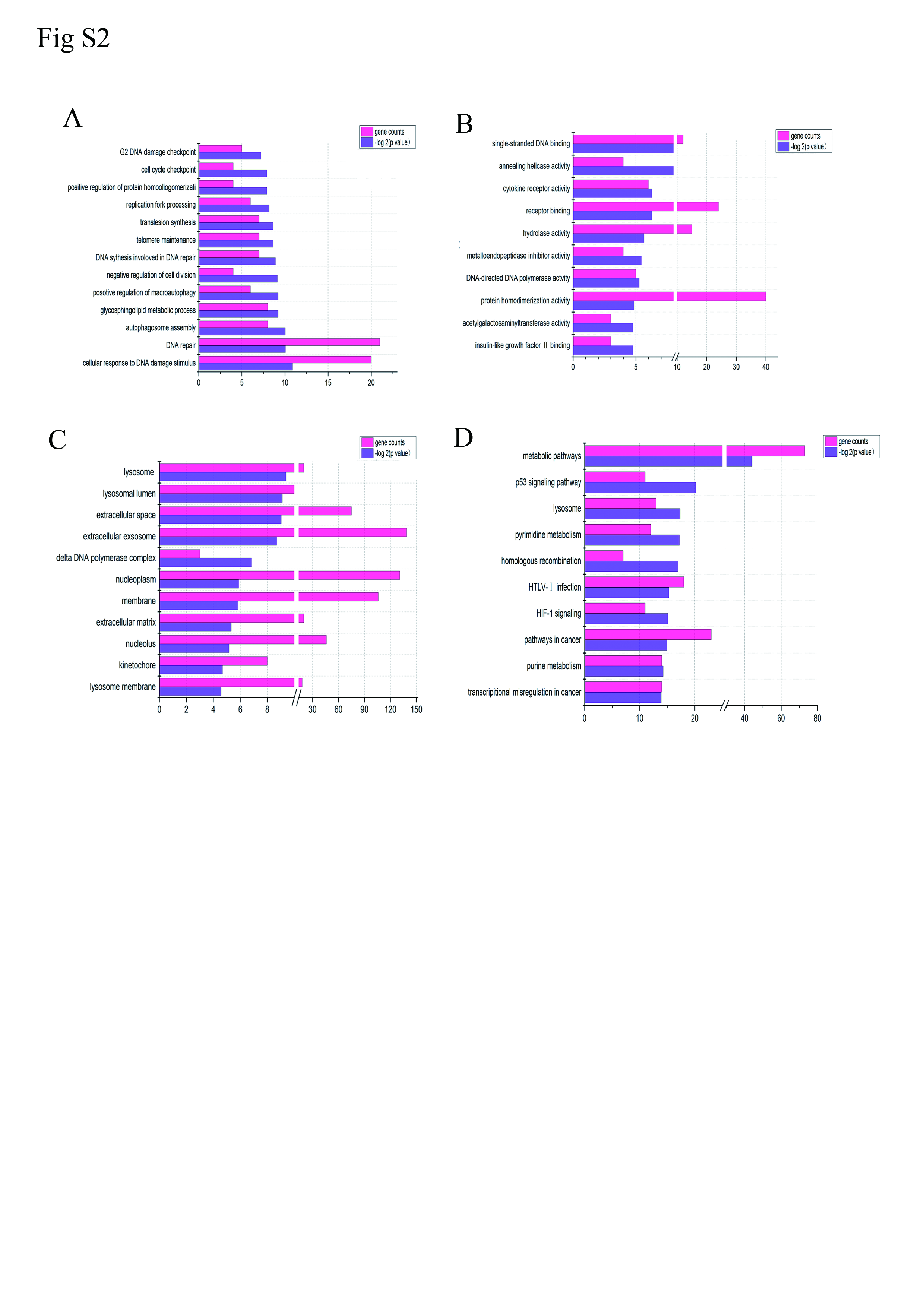

Supplement: Supplementary file 3 — Supplementary Figure S2 [file 41418_2018_141_MOESM3_ESM.tif]
