# Supplementary material for: The UbL-UBA Ubiquilin4 protein functions as a tumor suppressor in gastric cancer by p53-dependent and p53-independent regulation of p21
Source: Cell Death Differ. 2018 Jun 13;26(3):516–30. doi: 10.1038/s41418-018-0141-4 (PMC6370890; doi:10.1038/s41418-018-0141-4)
Supplement: Supplementary file 7 — Supplementary Table S4 [file 41418_2018_141_MOESM7_ESM.docx]

Supplementary Table S4 Fold changes of representative DEGs

| **DEGs** | | **Primer sequence** | **Fold Change** | |
| --- | --- | --- | --- | --- |
| **Protein Name** | **Gene Symbol** |  | **DNA microarray** | **Real-time PCR** |
| Metallothionein-4 | MT4 | F: 5’-GACCCCAGGGAATGTGTCT-3’ | 134.29 | 285.12 |
|  |  | R: 5’-GCAGCTCTTCCGACATGTTT-3’ |  |  |
| Interleukin-1 beta | IL1B | F: 5’-AGCTACGAATCTCCGACCAC-3’ | 8.85 | 14.14 |
|  |  | R: 5’-CGTTATCCCATGTGTCGAAGAA-3’ |  |  |
| E3 ubiquitin-protein ligase TRIM31 | TRIM31 | F: 5’-AACCTGTCACCATCGACTGTG-3’ | 8.74 | 10.71 |
|  |  | R: 5’-TGATTGCGTTCTTCCTTACGG-3’ |  |  |
| Eukaryotic translation initiation factor 2-alpha kinase 3 | EIF2AK3 | F: 5’-TCATCCAGCCTTAGCAAACC-3’ | 5.47 | 2.27 |
|  |  | R: 5’-ATGCTTTCACGGTCTTGGTC-3’ |  |  |
| Interleukin-13 receptor subunit alpha-2 | IL13RA2 | F: 5’-AAAGTTCAGGATATGGATTGCGT-3’ | 5.05 | 7.73 |
|  |  | R: 5’-GAAGTACACCTATGCCAGGTTTC-3’ |  |  |
| Cellular retinoic acid-binding protein 2 | CRABP2 | F: 5’-ATCGGAAAACTTCGAGGAATTGC-3’ | 3.72 | 3.10 |
|  |  | R: 5’-AGGCTCTTACAGGGCCTCC-3’ |  |  |
| Cyclin-dependent kinase inhibitor 1/p21 | CDKN1A | F: 5’-AGTCAGTTCCTTGTGGAGCC-3’ | 3.66 | 8.04 |
|  |  | R: 5’-CATGGGTTCTGACGGACAT-3’ |  |  |
| Peroxisomal acyl-coenzyme A oxidase 2 | ACOX2 | F: 5’-TCCACAGTTACCCGGAGTTTA-3’ | 2.92 | 1.94 |
|  |  | R: 5’-GCCGAGCTATCAACCGGAT-3’ |  |  |
| Glutaminase liver isoform, mitochondrial | GLS2 | F: 5’-GCAGAGAGACGCCACACAG-3’ | -7.57 | 0.91 |
|  |  | R: 5’-GGATTCGTTCCTGTCCTTCA-3’ |  |  |
| PHD finger protein 12 | PHF12 | F: 5’-ATCGTGTACGACTTGGACACA-3’ | -3.49 | 1.33 |
|  |  | R: 5’-CCACCTTCCTTGCAGCTATCG-3’ |  |  |
| Ribosomal protein S6 kinase alpha-5 | RPS6KA5 | F: 5’-AGGCAGTCGCCATTTTTGGTA-3’ | -3.08 | 1.19 |
|  |  | R: 5’-TCTCCAACATAAATCTGCACCTC-3’ |  |  |
| Neuron navigator 2 | NAV2 | F: 5’-ACTGGGCCAATCATTACCTAGC-3’ | -3.00 | 1.28 |
|  |  | R: 5’-CGCCATCTGTCACATCTTGCT-3’ |  |  |
| DEP domain-containing mTOR-interacting protein | DEPTOR | F: 5’-GCGGAGGCGAAGACTGATG-3’ | -2.76 | 0.39 |
|  |  | R: 5’-GGCTCACTGACATAAAGCTGGTA-3’ |  |  |
| Acrosin-binding protein | ACRBP | F: 5’-GCAGCCCAGGATTCGACTC-3’ | -2.06 | 0.84 |
|  |  | R: 5’-GTGCGAAGAAGCGTTCGTATT-3’ |  |  |
| Derlin-3 | DERL3 | F: 5’-GGTGAGGGTCAACTTCTTCG-3’ | -2.27 | 0.69 |
|  |  | R: 5’-TAGTAGATATGGCCCACCGC-3’ |  |  |
| ARF GTPase-activating protein GIT1 | GIT1 | F: 5’-GAGTCACCGCCAAAGACCTC-3’ | -2.17 | 0.71 |
|  |  | R: 5’-CAGCCCCATACACTACAAGCA-3’ |  |  |
| Y-box-binding protein 2SESN | YBX2 | F: 5’-AGAAGCCACTAATGTAACTGGGC-3’ | -2.14 | 0.52 |
|  |  | R: 5’-CGGGACTTACGTCGGTTGG-3’ |  |  |
| Cellular tumor antigen p53 | TP53 | F: 5’-CTTGGAACTCAAGGATGCCC-3’ | -1.22 | 1.16 |
|  |  | R: 5’-TTATGGCGGGAGGTAGACTGA-3’ |  |  |
| E3 ubiquitin-protein ligase Mdm2 | MDM2 | F: 5’-GAATCATCGGACTCAGGTACATC-3’ | 1.66 | 1.74 |
|  |  | R: 5’-TCTGTCTCACTAATTGCTCTCCT-3’ |  |  |
| Tumor necrosis factor receptor superfamily member 6 | FAS | F: 5’-TCTGGTTCTTACGTCTGTTGC-3’ | 1.63 | 1.27 |
|  |  | R: 5’-CTGTGCAGTCCCTAGCTTTCC-3’ |  |  |
| G1/S-specific cyclin-E2 | CCNE2 | F: 5’-TCAAGACGAAGTAGCCGTTTAC-3’ | -1.55 | 1.65 |
|  |  | F: 5’-GACCTCAACGCACAGTACGAG-3’ |  |  |
| Bcl-2-binding component 3 | BBC3/PUMA | R: 5’-AGGAGTCCCATGATGAGATTGT-3’ | 1.54 | 3.02 |
|  |  | F: 5S’-GGAGGACCGGAAAACCTCTAC-3’ |  |  |
| Quinone oxidoreductase PIG3 (PIGs) | TP53I3 | R: 5’-CCTCAAGTCCCAAAATGTTGCT-3’ | 1.65 | 1.79 |
|  |  | F: 5’-GTCAACGCTAGTGCCGTCAG-3’ |  |  |
| Insulin-like growth factor-binding protein 3/IGF-BP3 | IGFBP3 | R: 5’-CTTGGGATCAGACACCCG-3’ | -1.83 | 0.92 |
|  |  | F: 5’-ACCGCAACGTGGTTTTCTCA-3’ |  |  |
| Plasminogen activator inhibitor 1 | PAI/SERPINE1 | R: 5’-TTGAATCCCATAGCTGCTTGAAT-3’ | 2.13 | 3.42 |
|  |  | F: 5’-ACTGAAACTAATCAAGCGGCTC-3’ |  |  |
| Serpin B5 /Maspin | SERPINB5 | R: 5’-CTTTGCATACGGTCTCTTCGTAG-3’ | 1.61 | 2.87 |
|  |  | F: 5’-ATTGGGCCTTGCGATGGATAG-3’ |  |  |
| Ribonucleoside-diphosphate reductase subunit M2 B | P53R2/RRM2B | R: 5’-GAGTCCTGGCATAAGACCTCT-3’ | 1.66 | 2.68 |
|  |  | F: 5’-CTACATTGGAATAATGGCTGCGG-3’ |  |  |
| Sestrin-2 | SESN2 | F: 5’-CGCTCTCCTCCTTCGTGTT-3’ | 1.74 | 2.31 |
|  |  | R: 5’-CGCTCTCCTCCTTCGTGTT-3’ |  |  |
