# Supplementary material for: The UbL-UBA Ubiquilin4 protein functions as a tumor suppressor in gastric cancer by p53-dependent and p53-independent regulation of p21
Source: Cell Death Differ. 2018 Jun 13;26(3):516–30. doi: 10.1038/s41418-018-0141-4 (PMC6370890; doi:10.1038/s41418-018-0141-4)
Supplement: Supplementary file 9 — Supplementary Figure Legends [file 41418_2018_141_MOESM9_ESM.docx]

**Supplementary Fig S1 Establishment of stable cell lines and the effect of Ubqln4 on GES-1 cells**

(A) MKN45 and BGC-823 cells were infected with Ubqln4 lentivirus and empty lentivirus infected cells were used as controls. Cells were harvested and immunoblotted against Ubqln4 after 2 weeks of screening with 2 μg/ml puromycin. (B, C) Cell morphology (B) and flow cytometry (C) in GES-1 cells expressing Ubqln4. Changes of cell apoptosis in bright field and flow cytometric assay shows increase of cell apoptosis.

**Supplementary Fig S2** **Gene expression profile alterations after Ubqln4 overexpression and knockdown**

(A–C) Gene Ontology analysis of DEGs in Biological Process, Molecular Function and Cellular Component. Results are ordered by p-value and the top ten of each category are displayed. (D) KEGG pathway analysis of DEGs. Results are ordered by p-value and the top ten are displayed.

**Supplementary Fig S3** **CHX chase of c-MYC, EZH2 and ectopic RNF114 and p21 knockdown efficiency**

(A) MKN45 and BGC-823 cells were infected with Ubqln4-sh lentiviruses. GFP-sh lentivirus infected cells was used as controls. Cells were harvested and immunoblotted against Ubqln4 after 2 weeks of screening with 2 μg/ml puromycin. Ubqln4-sh1 and Ubqln4-sh2 were used in the following experiments. (B, C) Stable Ubqln4-expressing MKN45 cells and control MKN45 cells were treated with CHX (100 μg/ml) for (B) 0, 10, 20, 30, 40, 50 min or (C) 0, 0.5, 1, 2, 4, 6 or 8 h and cells were harvested and blotted against antibodies of (B) c-MYC or (C) EZH2. (D) MKN45 cells were infected with RNF114 lentivirus alone or together with Ubqln4 lentivirus. At 48 h after infection, cells were treated with CHX (100 μg/ml) for the indicated times and then harvested and immunoblotted for the indicated antibodies. (E, F) qRT-PCR and Western blot for p21 levels in MKN45 cells infected with various p21-shRNA lentiviruses.
